# Diverse novel resident *Wolbachia* strains in Culicine mosquitoes from Madagascar

**DOI:** 10.1038/s41598-018-35658-z

**Published:** 2018-11-29

**Authors:** Claire L. Jeffries, Luciano M. Tantely, Fara N. Raharimalala, Eliot Hurn, Sébastien Boyer, Thomas Walker

**Affiliations:** 1 Department of Disease Control, London School of Hygiene and Tropical Medicine, London, WC1E 7HT UK; 2 Institut Pasteur de Madagascar, Unité d’Entomologie Médicale, Ambatofotsikely, 101-Antananarivo, Madagascar; 3 Present Address: Medical Entomology Platform, Institut Pasteur of Cambodge, 5 Bd Monivong, PO Box 983, Phnom Penh, Cambodia

## Abstract

*Wolbachia* endosymbiotic bacteria are widespread throughout insect species and *Wolbachia* transinfected in *Aedes* mosquito species has formed the basis for biocontrol programs as *Wolbachia* strains inhibit arboviral replication and can spread through populations. Resident strains in wild Culicine mosquito populations (the vectors of most arboviruses) requires further investigation given resident strains can also affect arboviral transmission. As Madagascar has a large diversity of both Culicine species and has had recent arboviral outbreaks, an entomology survey was undertaken, in five ecologically diverse sites, to determine the *Wolbachia* prevalence. We detected diverse novel resident *Wolbachia* strains within the *Aedeomyia*, *Culex*, *Ficalbia*, *Mansonia* and *Uranotaenia* genera. *Wolbachia* prevalence rates and strain characterisation through Sanger sequencing with multilocus sequence typing (MLST) and phylogenetic analysis revealed significant diversity and we detected co-infections with the environmentally acquired bacteria *Asaia*. Mosquitoes were screened for major arboviruses to investigate if any evidence could be provided for their potential role in transmission and we report the presence of Rift Valley fever virus in three *Culex* species: *Culex tritaeniorhynchus*, *Culex antennatus* and *Culex decens*. The implications of the presence of resident *Wolbachia* strains are discussed and how the discovery of novel strains can be utilized for applications in the development of biocontrol strategies.

## Introduction

The endosymbiotic bacterium *Wolbachia* naturally infects approximately 40% of insect species^1^, including mosquito vector species that are responsible for transmission of human diseases. Resident *Wolbachia* strains are present in some major arbovirus disease vectors such as *Culex (Cx*.*) quinquefasciatus*^2–4^ and *Aedes (Ae*.*) albopictus*^5,6^. *Wolbachia* has been considered for mosquito biocontrol strategies due to the varied phenotypic effects strains have on host mosquito species. Some *Wolbachia* strains can induce a reproductive phenotype termed cytoplasmic incompatibility (CI). This phenotype results in inviable offspring when an uninfected female mates with a *Wolbachia*-infected male. In contrast, *Wolbachia*-infected females produce viable progeny when they mate with both infected and uninfected male mosquitoes. This reproductive advantage over uninfected females allows *Wolbachia* to invade mosquito populations. The CI phenotype was utilized in trials conducted in the late 1960s to eradicate *Cx*. *quinquefasciatus* from Myanmar^7^. Releasing large numbers of *Wolbachia*-infected male mosquitoes, to compete with wild type males to induce sterility, is known as the incompatible insect technique (IIT)^8,9^. Field trials have been undertaken using IIT for both *Ae*. *albopictus*^10^ and *Ae*. *polynesiensis*, a vector of lymphatic filariasis in the South Pacific^8^.

*Wolbachia* strains have also been shown to protect their native *Drosophila* fruit fly hosts against infection by pathogenic RNA viruses^11,12^. This discovery led to an alternative biocontrol approach for dengue virus (DENV) transmission which utilizes *Wolbachia*’s ability to inhibit pathogen replication within mosquitoes^13–17^. *Wolbachia* strains were successfully established in *Ae*. *aegypti*, the principle mosquito vector of DENV, yellow fever virus (YFV) and Zika virus (ZIKV), through embryo microinjection^15,16,18–21^. Preliminary field releases demonstrated the ability of the transinfected *w*Mel strain of *Wolbachia* to establish in wild mosquito populations^22^ and to provide strong inhibition of DENV in wild mosquitoes^23^. Recently it has been shown that *Wolbachia* frequencies have remained stable since deployment in an area of Townsville, Australia and to date no local dengue transmission has been confirmed after *Wolbachia* has established, despite local transmission events every year for the previous 13 years with a trend of increasing imported cases^24^. Further releases are ongoing in DENV endemic countries and mathematical models suggest the *w*Mel strain of *Wolbachia* could reduce the basic reproduction number, R0, of DENV transmission by 66–75%^25^. *Wolbachia* strains also inhibit other medically important arboviruses including chikungunya virus (CHIKV)^14,26^, YFV^27^ and recently ZIKV^26,28^.

There is also evidence that resident *Wolbachia* strains in laboratory mosquito colonies can inhibit arbovirus transmission^29–32^. Therefore, the prevalence and diversity of natural *Wolbachia* strains in field populations of mosquitoes requires further investigation to fully understand how widespread this bacterium is in wild Culicine populations. For example, recent studies undertaken to determine if *Ae*. *aegypti* contains resident strains report contrasting results with no evidence seen in a large collection from 27 countries^33^ but detection of a resident strain in populations from the Southeastern United States^34^. Additional bacterial species that make up the mosquito microbiota may influence the presence of resident *Wolbachia* strains. In *Ae*. *aegypti* laboratory colonies, the acetic acid bacterium *Asaia* infects the gut and salivary glands and there is competition with transinfected *Wolbachia* strains for colonisation of mosquito reproductive tissues^35^. *Asaia* has been detected in field populations of *Cx*. *quinquefasciatus*^36^ suggesting a complex association between these two bacterial species in wild mosquito populations.

The Culicinae subfamily of mosquitoes (Family Culicidae) contains a large variety of species (>3,000) particularly concentrated in tropical regions. Genera that transmit, or are implicated in transmitting, arboviruses include *Aedeomyia (Ad*.*)*, *Aedes*, *Culex*, *Mansonia (Ma*.*)* and *Uranotaenia* (*Ur*.*)*. Madagascar, a large island located off the southeast coast of mainland Africa, represents an optimal location to investigate *Wolbachia* prevalence in Culicines given its geographic isolation and large diversity of both mosquito species and arboviral diseases. Currently there have been 237 mosquito species morphologically identified in Madagascar in 15 different genera^37,38^, with 59% of these species thought to be endemic species in Madagascar. There have also been several arbovirus outbreaks in recent history including Rift Valley fever virus (RVFV)^39^, DENV (2006), CHIKV (2006, 2007, 2009 & 2010) and West Nile virus (WNV)^40^. Previous studies have identified 64 species in Madagascar that are implicated in transmission of medical or veterinary pathogens^37^. In this study, we undertook an entomology survey in five ecologically diverse sites in Madagascar to determine the prevalence of resident *Wolbachia* strains in adult female mosquitoes and discovered multiple novel *Wolbachia* strains. The characterisation, typing and phylogeny of these novel *Wolbachia* strains was analysed through Sanger sequencing, with multilocus sequence typing (MLST), to determine the diversity and evolutionary history of resident strains. We also compared *Wolbachia* and *Asaia* prevalence rates and screened mosquitoes for major medically important arboviruses to investigate if there was evidence for the involvement of any of these species as potential arbovirus vectors in these sites.

## Results

### Mosquito abundance and diversity at study sites

**Figure 1:**
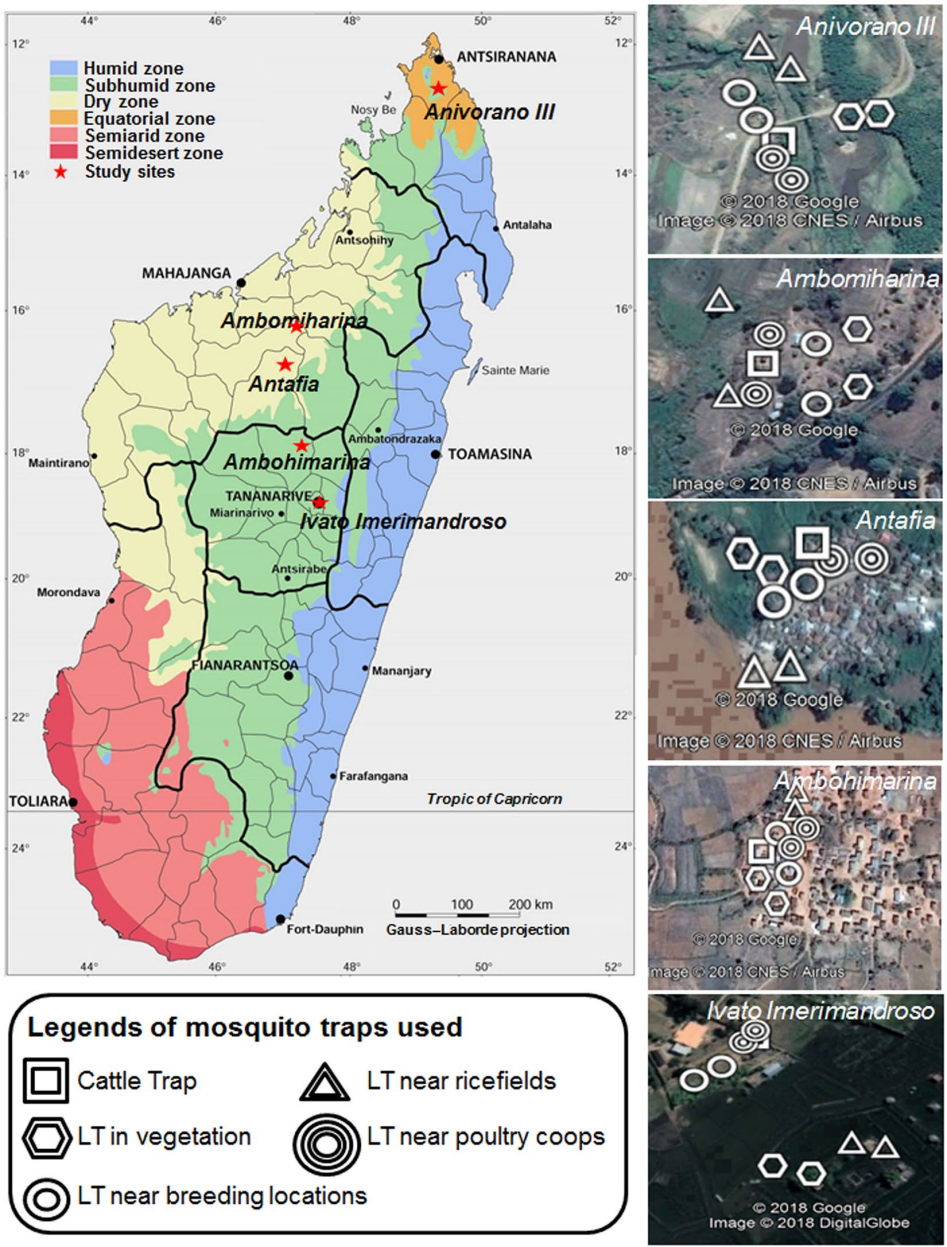
Map of Madagascar showing location of collection sites and mosquito traps used. Adult trapping was undertaken in five ecologically diverse sites using both CDC light traps (LT) and Cattle (zebu) baited traps (CT) near rice fields, poultry coups and breeding locations to maximize the diversity of species collected. Anivorano III is located within commune Anivorano Nord, Ambomiharina is located within commune Tsaramandroso, Antafia is located within commune Bemokotra, Ambohimarina is located within commune Ankazobe and Ivato Imerimandroso is located within commune Ivato Aeroport. Bioclimatic map of Madagascar adapted from Brunhes *et al*.^38^ using Microsoft Powerpoint (2010) and Paint Net (v4.0.6). Google Earth Pro (version 7.3) was used for satellite imagery as follows: *Source:* «Ivato Imerimandroso» 18°47′21.71″S and 47°28′42.34″E. Google Earth Pro. June 17, 2018. October 17, 2018. *Source:* «Ambomiharina.» 18°19′38.86″S and 47°06′23.91″E. Google Earth Pro. Novembre 10, 2016. October 17, 2018, *Source:* «Antafia» 17°01′26.31″S and 46°45′41.97″E. Google Earth Pro. April 02, 2016. October 17, 2018, *Source:* «Ambomiharina» 16°21′38.92″S and 46°59′20.90″E. Google Earth Pro. July 24, 2016. October 17, 2018, *Source:* «Anivorano III» 12°45′31.11″S and 49°14′13.08″E. Google Earth Pro. July 31, 2016. October 17, 2018.

Overall, 1174 individuals belonging to seven genera were captured from five collection sites (Fig. 1; Table 1). Qualitative collection site characteristics, median humidity and temperature and altitude are described in Supplementary Table S1. *Cx*. *antennatus* was collected in large numbers from Tsaramandroso and Ivato Aeroport and *Cx*. *univitattus* was the dominant species in Ankazobe. 820 (69.85%) of the mosquitoes were caught in CDC light traps, while 354 (30.15%) were caught with Zebu-baited traps. Tsaramandroso had the largest number of individuals caught (n = 644) and highest diversity, which may have been influenced by both a high median relative humidity (79%) and temperature (24 °C) for the collection period. In contrast, Bemokotra had the smallest number of individuals collected (n = 19) and this could have been influenced by a lower median relative humidity (50%) despite a median temperature of 26 °C. Ivato Aeroport had the lowest diversity of species collected in the study and this could possibly be due to a low median temperature (11 °C) despite a median relative humidity of 88%. *Aedes*, *Aedeomyia* and *Ficalbia (Fi*.*)* species were collected only at lower elevation sites (Tsaramandroso, Bemokotra) and *Culex* species were collected predominantly at low and high elevation sites (Tsaramandroso, Ankazobe and Ivato Aeroport). However, it should be noted that there are many more factors that influence mosquito species abundance and diversity.

**Table 1:** Summary of mosquito species abundance using morphological identification from five sites in Madagascar.

	Anivorano Nord		Tsaramandroso		Bemokotra		Ankazobe	Ivato Aeroport		Total
	CT	LT	CT	LT	CT	LT	LT	CT	LT	
*Aedeomyia furfurea*	0	0	0	2	0	0	0	0	0	2
*Aedeomyia madagascarica*	0	0	0	9	0	0	0	0	0	9
*Aedes aegypti*	0	0	0	0	0	2	0	0	0	2
*Aedes albocephalus*	0	0	0	1	0	0	0	0	0	1
*Aedes circumluteolus*	0	0	0	1	0	0	0	0	0	1
*Culex antennatus*	8	6	250	91	0	5	52	32	81	525
*Culex bitaeniorhynchus*	0	4	1	14	0	1	0	0	0	20
*Culex decens*	0	3	1	12	0	6	0	0	0	22
*Culex giganteus*	4	0	0	1	0	0	0	0	1	6
*Culex pipiens complex*	1	1	0	9	0	0	32	0	1	44
*Culex poicilipes*	0	0	2	17	0	2	1	4	0	26
*Culex tritaeniorhynchus*	0	0	25	18	0	0	0	0	0	43
*Culex univittatus*	2	6	17	95	0	5	250	0	1	376
*Ficalbia circumtestacea*	0	0	0	3	0	1	0	0	0	4
*Lutzia tigripes*	0	1	0	0	0	0	0	0	0	1
*Mansonia uniformis*	5	10	2	50	0	0	0	0	0	67
*Uranotaenia sp*.	0	5	0	23	0	3	0	0	0	31
Total	20	36	298	346	0	19	335	36	84	1174

Trapping was undertaken for two consecutive nights with CDC light traps (LT) and cattle (zebu-baited) (CT) traps. Trapping was undertaken between dusk and dawn and mosquitoes from CT traps were collected by mouth aspiration.

### Detection of resident *Wolbachia* strains and mosquito species identification

**Figure 2:**
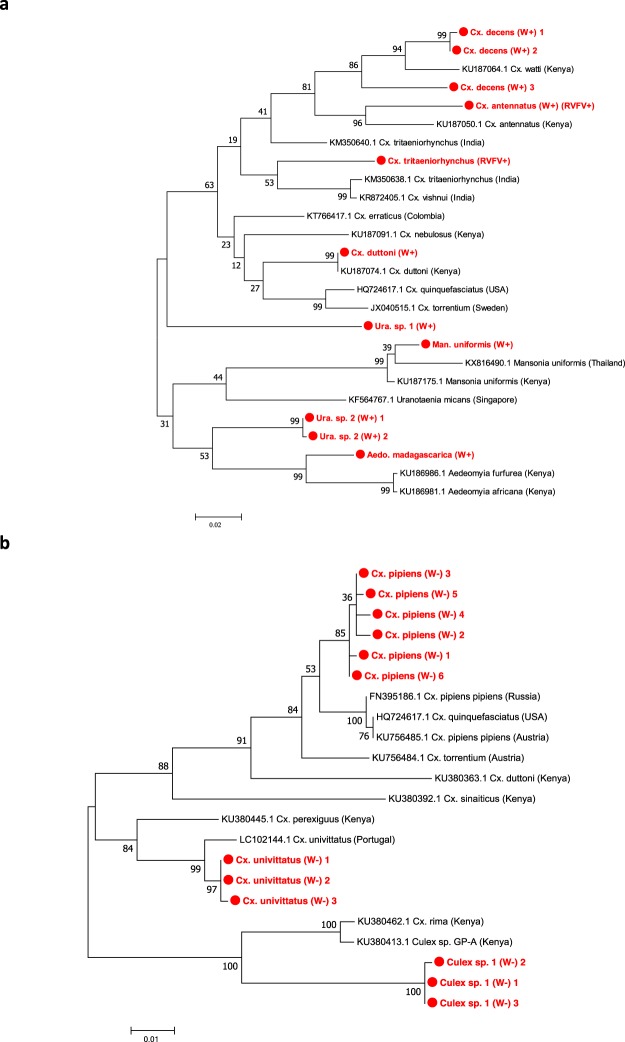
Madagascar mosquito species phylogenetic analysis using the CO1 gene. (**a**) Maximum Likelihood phylogenetic analysis of one fragment of the CO1 gene^41^ showing a tree with the highest log likelihood (−4419.95), comprising 26 nucleotide sequences. There were a total of 669 positions in the final dataset. (**b**) Maximum Likelihood phylogenetic analysis for selected *Culex* mosquito species targeting a second CO1 gene fragment^43^ showing a tree with the highest log likelihood (−2078.87), comprising 22 nucleotide sequences. There were a total of 638 positions in the final dataset. *Wolbachia*-infected = (W+), *Wolbachia*-uninfected = (W−), Rift Valley fever virus-infected = (RVFV+).

We screened individual mosquitoes for the presence of resident *Wolbachia* strains using three conserved *Wolbachia* genes (16 rRNA, wsp and ftsZ) and detected resident strains in six mosquito genera (Table 2). The prevalence of *Wolbachia* infection within mosquito species ranged from 3% (1/32) of *Cx*. *antennatus*, to 100% for *Ad*. *madagascarica* (9/9). PCR analysis resulted in congruency with the same individual samples amplifying fragments for all three genes, with the exception of *Ma*. *uniformis*, *Fi*. *circumtestacea*, *Cx*. *antennatus* and *Cx*. *duttoni*, in which the wsp gene was not amplified in any individuals. As Madagascar contains diverse mosquito species, we amplified a fragment of the mitochondrial cytochrome *c* oxidase subunit I (CO1) gene^41^ of *Wolbachia*-infected individuals to provide as much molecular confirmation of species as possible, given the lack of available CO1 sequences in certain regions of the gene for some species. We were unable to amplify any CO1 gene fragments of *Fi*. *circumtestacea* but the remaining *Wolbachia*-infected species were confirmed by successful CO1 sequencing (Fig. 2a). CO1 sequences were deposited in Genbank (accession numbers MK033247-MK033269, Supplementary Table S1) including the first CO1 sequences for *Ad*. *madagascarica*, and two *Uranotaenia* species from Madagascar, as well as the first *Cx*. *decens* sequences covering this region of the CO1 gene.

**Table 2:** *Wolbachia* and *Asaia* infection prevalence rates in adult female mosquitoes using three conserved *Wolbachia* genes (*16S rRNA*, *ftsZ* and *wsp*).

Mosquito species	Number	*Wolbachia* gene PCR amplification			Prevalence (%)		Fisher’s exact *post hoc* P value (*Wolbachia* vs. *Asaia*)
		*16S rRNA*	*ftsZ*	*wsp*	*Wolbachia*	*Asaia*	
*Aedes albocephalus*	1	0	0	0			
*Aedes circumlateolus*	1	0	0	0		100 (20–100)	
*Aedomyia furfuria*	2	0	0	0		100 (34–100)	
***Aedomyia madagascarica^***	**9**	**9**	**9**	**9**	**100** **(70–100)**	**89** **(56–98)**	>**0**.**99**
***Culex antennatus^***	**32**	**1**	**1**	**0**	**3** **(1–16)**	**56** **(38–74)**	**>0**.**99**
*Culex bitaeniorhynchus*	12	0	0	0		58 (32–81)	
***Culex decens^***	**17**	**3**	**3**	**3**	**18** **(4–43)**	**18** **(4–43)**	**0**.**46**
***Culex duttoni^***	**1**	**1**	**1**	**0**	**100** **(21–100)**	**100** **(21–100)**	**>0**.**99**
*Culex tritaeniorhynchus^*	19	0	0	0		95 (74–100)	
*Culex giganteus*	5	0	0	0		80 (37–96)	
*Culex pipiens complex*	44	0	0	0		7 (1–30)	
*Culex poicilipes*	21	0	0	0		48 (28–68)	
***Ficalbia circumtestacea***	**3**	**1**	**1**	**0**	**33** **(6–79)**		
***Mansonia uniformis^***	**24**	**7**	**7**	**0**	**29** **(15–49)**	**67** **(47–82)**	**0**.**65**
***Uranotaenia spp^***	**27**	**7**	**7**	**7**	**26** **(13–45)**	**19** **(8–37)**	**0**.**54**

An individual was considered *Wolbachia-*infected when any one of the three *Wolbachia* gene fragments were amplified. Species containing resident *Wolbachia* strains are in bold. *Wolbachia* and *Asaia* prevalence rates are shown with 95% confidence intervals in parentheses. Fisher’s exact *post hoc* test P value is shown comparing *Wolbachia* and *Asaia* infections in individual mosquitoes from species that contained at least one individual of both bacterial endosymbionts. ^ denotes where CO1 sequences were obtained for phylogenetic analysis of mosquito species.

Surprisingly, we found no evidence of resident *Wolbachia* strains in members of the *Cx*. *pipiens* complex indicating the absence of the *w*Pip strain which is usually ubiquitous in *Cx*. *pipiens pipiens* and *Cx*. *pipiens quinquefasciatus* sibling species^42^. As species within the *Cx*. *pipiens* complex are morphologically indistinguishable, we selected a sub-sample of individuals from different collection sites to determine the species using both species-specific PCR analysis and sequencing targeting a CO1 gene fragment that discriminates species within the *Cx*. *pipiens* complex^43^. PCR-based species identification targeting the *ace-2* gene^44^ revealed the presence of *Cx*. *pipiens pipiens* individuals in four collection sites (no *Cx*. *pipiens* complex individuals were collected from Bemokotra). Confirmation resulted from CO1 phylogenetic analysis and we confirmed six *Wolbachia*-uninfected *Cx*. *pipiens pipiens* within the *Cx*. *pipiens* complex (Fig. 2b**)**. We also analysed three females that were morphologically identified as *Cx*. *pipiens* complex individuals but did not result in any species confirmation through PCR (Fig. 2b). CO1 sequencing indicated these individuals were genetically diverse and, with the sequences currently available for comparison, were most closely related to *Cx*. *rima* and sequences named *Cx*. *sp*.*GP-A* from Kenya – Genbank accession number KU380413.1 (94% nucleotide identities, but only 89–94% coverage to the gene fragment amplified), suggesting the possibility of unknown *Culex* species present in Madagascar. We also screened individuals from all mosquito species for *Asaia* and detected the presence of this competing bacteria in 13 of 17 species (Table 2). Variable prevalence rates were observed and we detected co-infections of *Wolbachia* and *Asaia* in *Ad*. *madagascarica* (n = 8), *Cx*. *antennatus* (n = 1), *Cx*. *decens* (n = 2), *Cx*. *duttoni* (n = 1), *Ma*. *uniformis* (n = 5) and *Uranotaenia* species (n = 4). Fisher’s exact *post hoc* tests revealed no association between *Wolbachia* and *Asaia* prevalence rates in species that had at least one individual infected with either bacteria (Table 2).

### *Wolbachia* strain characterisations

**Figure 3:**
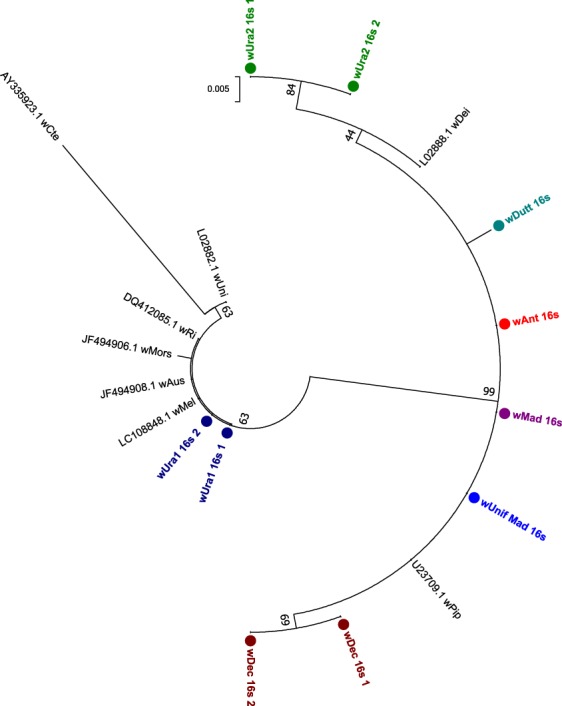
Maximum Likelihood phylogenetic analysis for novel *Wolbachia* strains using the 16S rRNA gene. The tree with the highest log likelihood (−681.38) is shown, comprising 17 nucleotide sequences. There were a total of 335 positions in the final dataset. Resident *Wolbachia* strains detected in our study are highlighted in colour, denoted with circles and multiple 16S rRNA sequences from individuals of the same species are included where possible. For comparison, a diverse range of strains available from GenBank were included (shown in black, with accession numbers included).

**Figure 4:**
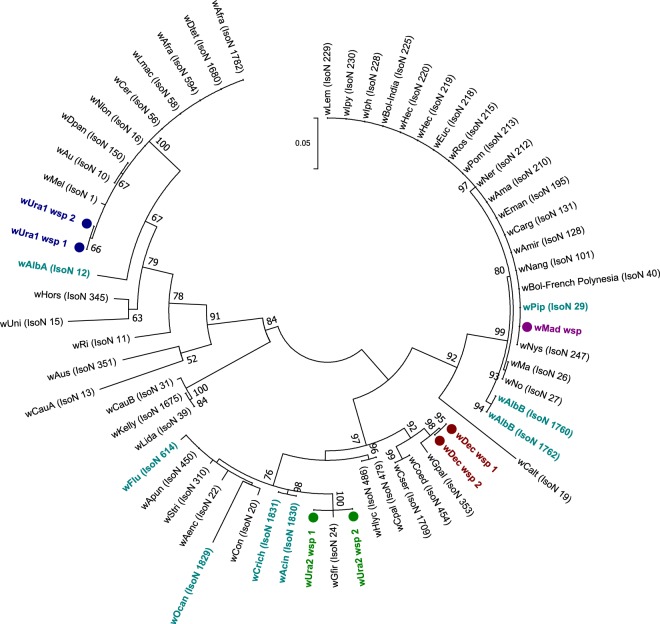
Maximum Likelihood phylogenetic analysis for novel *Wolbachia* strains using the wsp gene. The tree with the highest log likelihood (−2976.84) is shown, incorporating 62 nucleotide sequences. There were a total of 439 positions in the final dataset. Novel resident *Wolbachia* strains discovered and characterised in our study are highlighted in colour, denoted with circles and multiple wsp sequences from individuals of the same species are included where possible. Wsp sequence data from *Wolbachia* strains downloaded from the MLST database for comparison are shown in teal (mosquito strains) and black (all other host strains), with isolate numbers from the MLST database shown in brackets.

Sanger sequencing of 16S rRNA, wsp and multilocus sequence typing (MLST) gene fragments was undertaken with *Wolbachia*-infected individuals to characterise, type and determine strain phylogenies. As Madagascar contains some mosquito species that are only present on the island, we predicted the presence of divergent *Wolbachia* strains and this was reflected by variation in the success of amplifying gene target fragments using standard primers and alternative protocols that use M13 sequencing adaptors and/or primers comprising increased degeneracies^45^. Despite using a variety of primers, we were unable to amplify wsp or any of the five MLST gene loci for *Cx*. *antennatus* and *Cx*. *duttoni*. For the remaining resident *Wolbachia* strains, we amplified wsp from all strains except *w*Unif-Mad (Table 3). Phylogenetic analysis based on the 16S rRNA gene indicates most of these novel *Wolbachia* strains are clustering with Supergroup B *Wolbachia* strains, such as *w*Pip and *w*Dei (Fig. 3**)**. Interestingly, only *w*Ura1 clusters with Supergroup A *Wolbachia* strains, such as *w*Mel and *w*Ri. As the wsp gene has been evolving at a faster rate and provides more informative strain phylogenies, with improved phylogenetic resolution^46^, we compared the phylogenetic relationships for strains that amplified a wsp fragment (Fig. 4**)**. Similar phylogenetic relationships are shown with wsp, with *w*Ura1 sequences clustering with *w*Mel (Supergroup A), but the remaining strains (*w*Ura2, *w*Dec and *w*Mad) clustered with Supergroup B *Wolbachia* strains.

**Table 3:** Novel resident *Wolbachia* strain wsp and MLST gene allelic profiles.

Species	*Wolbachia* strain	*Wolbachia* gene allele					
		*wsp*	*gatB*	*CoxA*	*hcpA*	*ftsZ*	*fbpA*
*Ad*. *madagascarica*	*w*Mad	**10**	**4**	**14**	**40**	73*	**4**
*Uranotaenia spp 1*	*w*Ura1	*538* *(2)*	**53**	**50**	*67* (*3*)	*55* *(1)*	**61**
*Uranotaenia spp 2*	*w*Ura2	*148* *(4)*	*9* *(1)*	*68* *(1)*	*6* *(1)*	**7**	**10**
*Ma*. *uniformis*	*w*Unif-Mad	—	**9**	**14**	—	**73**	*4* *(2)*
*Cx*. *decens*	*w*Dec	*561* *(13)*	**9**	*14* *(2)*	*12* *(1)*	*9* *(2)*	*203* *(1)*
*Fi*. *circumtestacea*	*w*Cir	—	219 *(2)*	*36* *(2)*	*40* *(1)*	*81* *(1)*	**219**

Exact matches to existing alleles present in the database are shown in bold, novel alleles are shown in italics and denoted by the allele number of the closest match (number of single nucleotide differences to the closest match). *Alternative degenerate primers used to generate sequence.

MLST resulted in successful sequencing of the majority of gene loci, although we were unable to amplify hcpA from *w*Unif-Mad despite using alternative protocols with degenerate primers (Table 3). ftsZ universal primers^47^ were also needed to obtain a sequence for *w*Mad. Each of the *w*Mad gene sequences produced an exact match to alleles already present in the database, with the resulting allelic profile producing an exact match with strain type 125. This allelic profile matches four isolates currently present in the MLST database, detected in three Lepidopteran species, from French Polynesia, India, Japan and Tanzania (Isolate numbers 40, 247, 270 and 322 respectively), but no isolates from mosquito or other Dipteran hosts. The remaining strains had new alleles for at least one of the MLST gene loci (sequences differed from those currently present in the database by at least one nucleotide difference per locus), resulting in new allelic profiles highlighting the diversity and novelty of these resident strains. *Wolbachia* gene sequences were deposited in GenBank (accession numbers MK026554-MK026563 and MK033270-MK033320, Supplementary Table S2).

**Figure 5:**
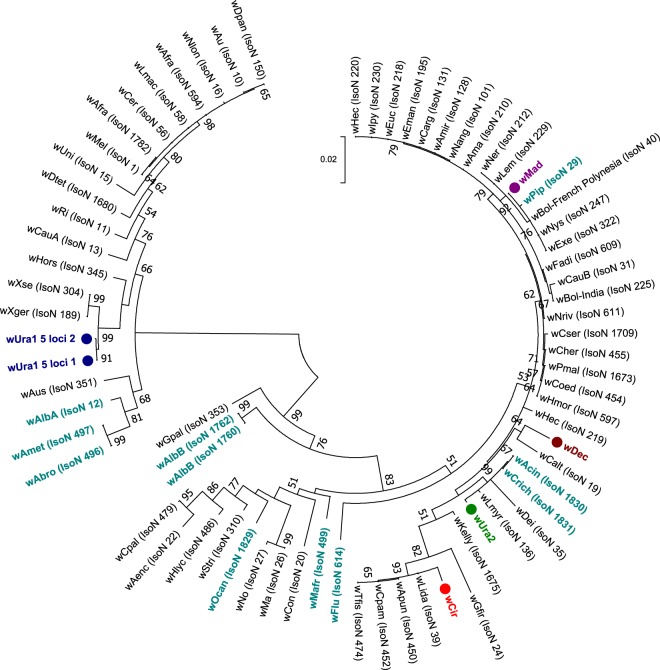
Maximum Likelihood molecular phylogenetic analysis from concatenation of the five MLST loci. The tree with the highest log likelihood (−9010.45) is shown incorporating 73 nucleotide sequences. There were a total of 2063 positions in the final dataset. Novel resident strains discovered and characterised in our study are highlighted in colour and denoted with circles. Concatenated sequence data from *Wolbachia* strains downloaded from the MLST database for comparison are shown in teal (mosquito strains) and black (all other host strains), with isolate numbers from the MLST database shown in brackets.

**Figure 6:**
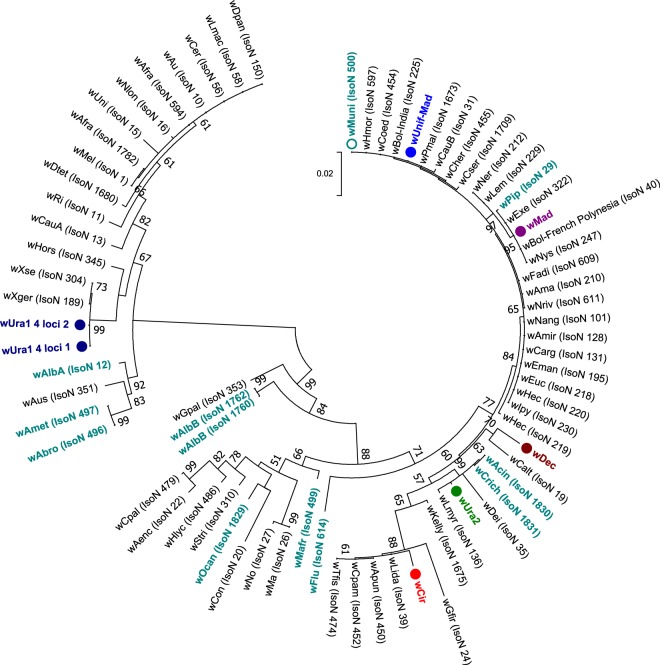
Maximum Likelihood phylogenetic analysis from concatenation of four MLST loci (coxA, fbpA, ftsZ and gatB). The tree with the highest log likelihood (−7114.64) is shown incorporating 75 nucleotide sequences. There were a total of 1624 positions in the final dataset. Concatenated sequence data from *Wolbachia* strains downloaded from the MLST database for comparison are shown in teal (mosquito strains) and black (all other host strains), with isolate numbers from the MLST database shown in brackets. Concatenated *Ma*. *uniformis* sequence data from Kenya^60^ (Isolate number 500) downloaded from the MLST database is highlighted in teal and denoted with a clear circle, for comparison with the *w*Unif-Mad strain (blue with filled circle) detected in this study. Other novel resident strains discovered and characterised in our study are highlighted in colour and denoted with filled circles.

The phylogeny of these novel strains based on concatenated sequences of all five MLST gene loci (where possible) **(**Fig. 5**)**, or four MLST gene loci for analysis of *w*Unif-Mad (hcpA sequence not available) alongside the other strains **(**Fig. 6**)**, also reveals significant strain divergence. As expected, due to the general incongruence between host phylogenetic relationships and their resident *Wolbachia* strains^48,49^, the majority of the novel *Wolbachia* strains in our study provide no evidence for clustering with other strains that infect mosquitoes. One exception is the *w*Mad strain which, although distinct, appears closely related to *w*Pip (the resident strain normally present in the *Cx*. *pipiens* complex). The other exception is the *w*Unif-Mad strain (based on 4 MLST loci, without hcpA), which is closely related to the *Wolbachia* strain previously detected in *Ma*. *uniformis* in Kenya (Isolate number 500), and where the pattern of amplification success was also matched as there was absence of amplification for both hcpA and wsp. However, *w*Unif-Mad differs by two nucleotides within the fbpA sequence, producing a novel fbpA allele and therefore a new allelic profile **(**Fig. 6 and Table 3**)**.

### Site locations for *Wolbachia*-infected mosquitoes

The novel resident *Wolbachia* strain in *Cx*. *decens*, named *w*Dec, was only found in individuals collected from Tsaramandroso with no detectable infections in individuals from Anivorano Nord or Bemokotra. Resident *Wolbachia* strains in both *Cx*. *antennatus* (*w*Ant) and *Cx*. *duttoni* (*w*Dut) were from individuals collected in Tsaramandroso. The novel *w*Mad *Wolbachia* strain was detected in all *Ad*. *madagascarica* females collected in Tsaramandroso. We detected the *w*Unif-Mad strain in *Ma*. *uniformis* from both Tsaramandroso and Anivorano Nord. Our single *Wolbachia*-infected *Fi*. *circumtestacea* (*w*Cir) was collected from Tsaramandroso and novel *Wolbachia* strains in *Uranotaenia* species (*w*Ura1 and *w*Ura2) were detected in individuals from Anivorano Nord, Tsaramandroso and Bemokotra.

### Arbovirus detection in mosquitoes

In total, 704 mosquitoes of species considered potential vectors of medically important arboviruses in Madagascar were screened using arbovirus-specific PCR assays. Mosquitoes were screened as either individuals or pools of 3–5 individuals from the same location and trapping method (Table 1). RVFV RNA was detected in two individually screened female adults of *Cx*. *decens*: one collected from Tsaramandroso and one from Bemokotra. We also detected RVFV in ten individual *Cx*. *tritaeniorhynchus* females and five individual *Cx*. *antennatus* females from Tsaramandroso. We sequenced RVFV PCR products from all positive individuals to confirm correct target amplification and also used CO1 sequencing to confirm mosquito species (representative samples are shown in Fig. 2a). No evidence of infection for the remaining arboviruses was present from any mosquitoes collected from the five sites in our study.

## Discussion

Madagascar contains some of the most diverse and unique flora and fauna given its geographical isolation. There are currently 237 known species of mosquitoes present on the island, but recent newly described species^37^ would suggest the possibility of more species present only in Madagascar. In our study, we assessed the *Wolbachia* prevalence of mosquito species that have the potential to be arbovirus vectors from five diverse ecological sites. To our knowledge, we are reporting for the first time resident *Wolbachia* strains in seven mosquito species: *Ad*. *madagascarica*, *Cx*. *antennatus*, *Cx*. *decens*, *Cx*. *duttoni*, *Fi*. *circumtestacea* and two *Uranotaenia* species. A limitation of this study is that sampled mosquito species were collected at night preventing an analysis of resident *Wolbachia* strains present in day-active mosquitoes. Further studies should be undertaken in these sites using mosquito traps such as Biogents (BG) sentinel traps that target the collection of species more active during the day such as *Ae*. *aegypti* and *Ae*. *albopictus*.

The novel resident *Wolbachia* strain in *Cx*. *decens*, named *w*Dec, was only found in individuals collected from one location (Tsaramandroso) and an overall prevalence of 20% indicates that prevalence rates are likely to be variable across the island. *Cx*. *decens* is present in all biogeographic domains of Madagascar and is particularly abundant in the central domain^50^. In Madagascar, this species was previously shown to be infected with WNV and Babahoyo virus^50^ and in mainland Africa has been shown to be infected with additional arboviruses including Moussa virus^51^. The *w*Mad strain was found in all *Ad*. *madagascarica* individuals we collected from Tsaramandroso. *Ad*. *madagascarica* was previously found naturally infected with WNV and is zoophilic, with a preference for feeding on avian blood^52^, demonstrating its likely involvement in zoonotic arbovirus transmission in Madagascar. In previous entomological surveys in north-western Madagascar where WNV is endemic, *Ad*. *madagascarica* was the most abundant species and WNV RNA was detected in one pool suggesting this species may play a role in WNV maintenance or transmission^53^.

Novel resident *Wolbachia* strains were also detected in two *Uranotaenia* species, of which there is very limited knowledge of these species in Madagascar. The lack of available CO1 sequences prevented molecular species confirmation but *Ur*. *anopheloides* is endemic to Madagascar and the Comoros archipelago and is most abundant is warmer regions of the western domain^38^. *Ur*. *alboabdominalis* is thought to occur mainly in the eastern and western domains of Madagascar and *Ur*. *neireti* in the central and eastern domains between 900–2000 m above sea level^37^.

There is also limited knowledge of *Fi*. *circumtestacea* in Madagascar, with reports of its presence in the eastern and western domains^37^, but this species is not currently confirmed as being involved in arbovirus transmission. As we were only able to amplify a fragment of the 16S rRNA gene for resident strains in *Cx*. *antennatus* (*w*Ant) and *Cx*. *duttoni* (*w*Dut), further studies are needed to determine if the lack of additional *Wolbachia* gene amplification is due to strain variability or low-density infections preventing successful amplification. An alternative explanation could be that amplification resulted from *Wolbachia* genes integrated into the mosquito host genomes resulting from ancient horizontal gene transfers^54,55^. Although most genes transferred between *Wolbachia* and their hosts are non-functional in the recipient genome^56^, expression of genes associated with *Wolbachia* prophage regions was previously observed in *Ae*. *aegypti*^55^. As we extracted RNA from field caught mosquitoes, we were able to demonstrate expression of multiple *Wolbachia* genes (including the ftsZ cell cycle gene that plays a central role during bacterial cytokinesis and the *Wolbachia* surface protein gene for most strains) which would indicate amplification is more likely of bacterial gene origin rather than integrated into the host genome.

*Ma*. *uniformis* is present in all biogeographical domains of Madagascar, is considered an anthropophilic vector of numerous arboviruses such as RVFV^57^ and WNV^52^, and is a known vector of *Wuchereria bancrofti* filarial nematodes in numerous countries. Resident *Wolbachia* strains in *Ma*. *uniformis* have previously been shown in southeast Asia^58^, Kenya^59^ and more recently in Sri Lanka^60^. Interestingly in Sri Lanka, 3/3 (100%) of *Ma*. *uniformis* individuals amplified the wsp gene^60^ but in our study all seven individuals failed to amplify wsp. *Ma*. *uniformis* was also one of the four *Wolbachia*-infected mosquito species from populations in Kenya^59^ and this provides the only comparative MLST and reports the requirement for nested PCR to amplify hcpA. Our *Wolbachia* prevalence rate of 29% (7/24) for *Ma*. *uniformis* is similar to the 29% (5/19) reported in Kenyan populations^59^ suggesting this species has variable prevalence rates in wild populations.

The absence of *Wolbachia* infections in the *Cx*. *pipiens* complex (particularly *Cx*. *pipiens pipiens* which were confirmed by PCR and CO1 sequencing) was surprising given high infection rates of the *w*Pip strain are often observed in wild populations^61–65^. Furthermore, our results contrast with a study undertaken in Madagascar as part of a wider Incompatible Insect Technique (IIT) from southwestern Indian Ocean islands in which the *w*Pip strain was detected in *Cx*. *pipiens quinquefasciatus* mosquitoes from Antananarivo^66^. This suggests the possibility of variable infection rates between *Cx*. *pipiens pipiens* and its sibling species *Cx pipiens quinquefasciatus* in Madagascar populations (which we did not collect in our study). We also undertook molecular identification of three *Culex* individuals (also *Wolbachia*-uninfected) that were phylogenetically similar to *Cx*. *rima* (Fig. 2b) which is consistent with a previous entomological survey that describes an unknown species morphologically similar to *Cx*. *rima*^67^. Further phylogenetic studies are warranted to determine the diversity of *Culex* species and the variability of *Wolbachia* prevalence within the *Cx*. *pipiens* complex.

The presence of resident *Wolbachia* strains in mosquito vector species which can transmit human arboviruses could be influencing arboviral transmission dynamics in field populations of Madagascar. In laboratory studies, resident *Wolbachia* strains have been shown to impact arboviral transmission. For example, *Wolbachia* was shown to reduce DENV infection of salivary glands and limit transmission in *Ae*. *albopictus*^29^. Outbreaks of RVFV in Madagascar are thought to result from infected domestic animals imported from east Africa^68^ and *Cx*. *antennatus* has previously been identified as an important RVFV vector in Madagascar^39^. Our results would indicate *Cx*. *tritaeniorhynchus* is also likely contributing to transmission in Tsaramandroso given we also detected RVFV RNA in multiple non-blood fed females of this species. RVFV has not previously been detected in this species in Madagascar, however, *Cx*. *tritaeniorhynchus* was implicated as a major vector during a large outbreak of RVFV in Saudi Arabia^69^ so this species contribution to transmission in Madagascar could potentially be currently underestimated.

The detection of RVFV in three *Culex* species at Tsaramandroso and *Cx*. *decens* at Bemokotra would correlate with previous studies that have shown that RVFV is one of the most abundant and widely distributed arboviruses across the island and there have been several recent RVFV outbreaks^39,70^. In our study, we detected RVFV in *Wolbachia-*uninfected *Cx*. *decens* and *Cx*. *tritaeniorhynchus* individuals but our single *Wolbachia*-infected *Cx*. *antennatus* was co-infected with RVFV. This female was non-blood fed indicating potential dissemination of RVFV beyond the bloodmeal. We were also unable to amplify any *Wolbachia* genes other than 16S rRNA from this individual which could be explained by a very low-density resident strain present in *Cx*. *antennatus* potentially resulting in the low prevalence rate of 3.1% (1/32). *Wolbachia* tissue tropism influences the potential to inhibit arboviruses^15,71^ as some resident *Wolbachia* strains are present predominantly in reproductive tissue and have little effects on arboviruses^72^ but transinfected strains that are present in tissues such as salivary glands have strong inhibitory effects^15,16^. However, as arbovirus infection rates are normally low in mosquito populations, correlating the prevalence of *Wolbachia* and RVFV would require a significantly larger number of mosquitoes.

The co-detection of resident *Wolbachia* strains and *Asaia* in seven species also indicates the potential for complex tripartite interaction with arboviruses. In our study, *Asaia* was also detected in the single *Wolbachia*-infected *Cx*. *antennatus* individual so further studies should be undertaken to determine the dynamics of these microbes within wild mosquito populations. The prevalence of *Asaia* is highly dependent on the environment given this bacterium can be acquired through various routes including from males to females during mating and from both larval and adult mosquito sugar feeding^73^. For example, prevalence rates of 46% were seen in field-caught *Ae*. *albopictus* from Madagascar^74^ but the overall composition and diversity of the mosquito microbiota in field populations of *Ae*. *aegypti* and *Ae*. *albopictus* in Madagascar is also highly dependent on environmental factors^75,76^. It has also recently been shown that there is no evidence for *Wolbachia-Asaia* co-infections in a wide range of *Anopheles* species and the environment likely influences *Asaia* prevalence and density in wild mosquito populations^77^.

The discovery of novel resident *Wolbachia* strains in mosquito species from Madagascar may also impact future attempts to extend *Wolbachia* biocontrol strategies by using these strains for applied use through transinfection. The naturally occurring *w*AlbA and *w*AlbB strains of *Wolbachia* that infect *Ae*. *albopictus* have been successfully transferred to *Ae*. *aegypti*^16,18,20^ and significantly inhibit DENV^16,20^. This suggests the introduction of novel resident *Wolbachia* strains from other Culicine species may generate inhibitory effects on arboviruses although this will be dependent on several factors and there is variability between *Wolbachia* strains on the strength of inhibition^15,16,20,21^. Further experiments should elucidate both the density and tissue tropism of these novel strains to determine candidate strains for transfer to species such as *Cx*. *quinquefasciatus* that are major arbovirus vectors. As *Cx*. *quinquefasciatus* contain a resident *Wolbachia* strain (*w*Pip)^78^, introduction of transinfected strains would require a stable association, with introduced strains growing to higher densities in specific tissues which result in inhibition of pathogen transmission^71^. Our discovery of novel resident strains in *Culex* species closely related to *Cx*. *quinquefasciatus* may improve transinfection success and ultimately lead to *Wolbachia* biocontrol strategies targeting *Culex* species.

## Methods

### Study sites

Five study sites were chosen according to a large variety of potential ecological factors in order to sample a wide variety of mosquito species (Fig. 1, Supplementary Table S3). Sampling was undertaken in June 2016 from one village within each of the following communes: Anivorano Nord (located in the Northern domain), Tsaramandroso and Bemokotra (Western domain), Ankazobe and Ivato Aeroport (Central domain). The village of Anivorano III (12°45′52.2″S, 49°14′19.3″E, at 375 m above sea level (asl)) is within the district of Antsiranana rural and is located 500 m south of the city of Anivorano Nord. The village consists of 32 houses distributed on both sides of the Irodo River. This river irrigates rice fields with heterogeneous rice phenology that are restricted to a small valley floor. The village of Ambomiharina (16°21′62.2″S, 46°59′34.4″E, at 84 m asl) is located in the Ambato-Boeny district within the Tsaramandroso commune. The village consists of 35 houses and is located 5 km south east of Ankarafantsika Forest, surrounded by a large rice field, irrigated by numerous large streams. The village of Antafia (17°01′37.8″S, 46°45′61.7″E, at 64 m asl) is located on the Betsiboka River, at 11 km south west of the city of Maevatanana (commune Bemokotra) and consists of 193 houses. The landscape consists of Betsiboka River in the west and large dry rice fields in the south, east and north of the village. Additionally, this site is characterized by a resident population of bats that were found both in houses and flying during dusk. The village of Ambohimarina (18°19′58.6″S, 47°′06.33.3″E, at 1212 m asl) is 5 km south of the city of Ankazobe (commune Ankazobe). The village consists of 289 houses, is located on a hill and surrounded with valleys composed of rice fields. The village of Ivato Imerimandroso (18°47′31.6″S, 47°28′68.4″E, at 1261 m asl) is located 300 m from Ivato Airport in the city of Antananarivo (commune Ivato Aeroport). Rice fields are flooded and surrounded with swamp and marshes. The domestic animals and livestock consist of cattle, dogs, and poultry in all five study sites.

### Mosquito sampling

Mosquito sampling was undertaken utilizing both CDC light traps and a net trap baited with Zebu (local species of cattle) to attract zoophilic species at each of the five study sites^70^. Trapping was performed over two consecutive nights per site, with CDC light traps set up at dusk to be operational during sunset and overnight, before being collected at dawn. Eight CDC light traps were placed around each site nearby dwellings, near poultry coops, inside areas of denser vegetation (typically forested areas or crop fields) and near potential breeding locations which varied depending on each site. At each site, one Zebu trap was placed in a way which simulated local animal husbandry practices (i.e. inside of a shed, nearby to a large corral, or on its own tied to a post). Zebu traps were set up following sunset and mosquitoes were collected by mouth aspiration before dawn. Temperature and relative humidity were recorded at each site using Lascar Electronics data loggers and the median was calculated over the collection period.

### Morphological identification and RNA extraction

Mosquitoes were anesthetized with chloroform vapor and morphological identification was performed using a variety of mosquito genera keys^37^. Following identification, mosquitoes were placed into 96 well storage plates or Eppendorf tubes and RNAlater (Sigma, UK) was added prior to storage at 4 °C or lower to prevent viral RNA degradation. RNA was extracted from individual whole mosquitoes (or additional pools for further detection of human pathogens) using QIAGEN RNeasy 96 kits according to manufacturer’s instructions. RNA was eluted in 40 μl of RNase-free water and stored at −80 °C. A QIAGEN QuantiTect Reverse Transcription kit was used to reverse transcribe RNA, generating cDNA from all RNA extracts, according to manufacturer’s instructions.

### Molecular species identification

Sanger sequencing targeting a fragment of the mitochondrial gene cytochrome oxidase 1 (CO1) gene was undertaken on mosquito cDNA extracts to confirm species identification^41^. Selected samples morphologically identified as part of the *Cx*. *pipiens* complex were further analyzed using multiplex PCR targeting the ace-2 locus^44^. Sanger sequencing was also undertaken targeting a different fragment of the CO1 gene specifically shown to provide the optimal discrimination of species within the *Culex pipiens* complex^43^.

### *Wolbachia* screening

*Wolbachia* detection was first undertaken targeting three conserved *Wolbachia* genes previously shown to amplify a wide diversity of strains; 16S rDNA gene^79^, *Wolbachia* surface protein (wsp) gene^46^ and FtsZ cell cycle gene^80^. DNA extracted from a *Drosophila melanogaster* fruit fly (infected with the *w*Mel strain of *Wolbachia*) was used as a positive control, in addition to no template controls (NTCs). 16S rDNA and wsp gene PCR reactions were carried out in a Bio-Rad T100 Thermal Cycler using standard cycling conditions^46,79^ and PCR products were separated and visualised using 2% E-gel EX agarose gels (Invitrogen) with SYBR safe and an Invitrogen E-gel iBase Real-Time Transilluminator. FtsZ gene real time PCR reactions were prepared using 5 µl of FastStart SYBR Green Master mix (Roche Diagnostics), a final concentration of 1 µM of each primer, 1 µl of PCR grade water and 2 µl template DNA, to a final reaction volume of 10 µl. Prepared reactions were run on a Roche LightCycler® 96 System for 15 minutes at 95 °C, followed by 40 cycles of 95 °C for 15 seconds and 58 °C for 30 seconds. Amplification was followed by a dissociation curve (95 °C for 10 seconds, 65 °C for 60 seconds and 97 °C for 1 second) to ensure the correct target sequence was being amplified. PCR results were analysed using the LightCycler® 96 software (Roche Diagnostics). *Asaia* detection was also undertaken targeting the 16S rRNA gene^73,81^.

### *Wolbachia* MLST

Multilocus sequence typing (MLST) was undertaken to characterize *Wolbachia* strains using the sequences of five conserved genes as molecular markers to genotype each strain. In brief, 450–500 base pair fragments of the coxA, fbpA, hcpA, gatB and ftsZ *Wolbachia* genes were amplified from one individual from each *Wolbachia*-infected mosquito species using previously optimised protocols^45^. A *Culex pipiens* gDNA extraction (previously shown to be infected with the *w*Pip strain of *Wolbachia*) was used a positive control for each PCR run, in addition to no template controls (NTCs). If no amplification was detected using standard primers, further PCR analysis was undertaken using alternative protocols which included utilisation of M13 sequencing adaptors and/or primers with increased degeneracies^45^.

### Sanger sequencing

PCR products were separated and visualised using 2% E-gel EX agarose gels (Invitrogen) with SYBR safe and an Invitrogen E-gel iBase Real-Time Transilluminator. PCR products were submitted to Source BioScience (Source BioScience Plc, Nottingham, UK) for PCR reaction clean-up, followed by Sanger sequencing to generate both forward and reverse reads. Sequencing analysis was carried out in MEGA7^82^ as follows. Both chromatograms (forward and reverse traces) from each sample was manually checked, analysed, and edited as required, followed by alignment by ClustalW and checking to produce consensus sequences. Consensus sequences were used to perform nucleotide BLAST (NCBI) database queries, and *Wolbachia* gene loci sequences were used for searches against the *Wolbachia* MLST database (http://pubmlst.org/wolbachia)^83^. If a sequence produced an exact match in the MLST database, we assigned the appropriate allele number.

### Phylogenetic analysis

Maximum Likelihood phylogenetic trees were constructed from Sanger sequences as follows. The evolutionary history was inferred by using the Maximum Likelihood method based on the Tamura-Nei model^84^. The tree with the highest log likelihood in each case is shown. The percentage of trees in which the associated taxa clustered together is shown next to the branches. Initial tree(s) for the heuristic search were obtained automatically by applying Neighbor-Joining and BioNJ algorithms to a matrix of pairwise distances estimated using the Maximum Composite Likelihood (MCL) approach, and then selecting the topology with superior log likelihood value. The trees are drawn to scale, with branch lengths measured in the number of substitutions per site. Codon positions included were 1st + 2nd + 3rd + Noncoding. All positions containing gaps and missing data were eliminated. The phylogeny test was by Bootstrap method with 1000 replications. Evolutionary analyses were conducted in MEGA7^82^.

### Arbovirus screening

Arbovirus screening using published PCR assays included the major arboviruses of public health importance, suspected or having the potential of being transmitted in Madagascar (Supplementary Table S4). PCR reactions for all assays except ZIKV were prepared using 5 µl of Qiagen SYBR Green Master mix, a final concentration of 1 µM of each primer, 1 µl of PCR grade water and 2 µl template cDNA, to a final reaction volume of 10 µl. Prepared reactions were run on a Roche LightCycler® 96 System and PCR cycling conditions are described in Supplementary Table S1. Amplification was followed by a dissociation curve (95 °C for 10 seconds, 65 °C for 60 seconds and 97 °C for 1 second) to ensure the correct target sequence was being amplified. ZIKV screening was undertaken using a Taqman probe-based assay using 5 µl of Qiagen QuantiTect probe master mix, a final concentration of 1 µM of each primer, 1 µl of PCR grade water and 2 µl template cDNA, to a final reaction volume of 10 µl. PCR results were analysed using the LightCycler® 96 software (Roche Diagnostics). Synthetic long oligonucleotide standards (Integrated DNA Technologies) of the amplified PCR product were generated in the absence of biological virus cDNA positive controls and each assay included negative (no template) controls.

### Statistical analysis

Fisher’s exact *post hoc* test in Graphpad Prism 6 was used to compare *Wolbachia* and *Asaia* prevalence rates in species that had at least one individual infected with *Wolbachia*.

### Ethics approval and consent to participate

Ethical clearance for the use of Zebu cattle in baited traps was obtained from the ethical committee of the Livestock ministry of Madagascar which is the sole relevant authority for animal care in Madagascar. The ethical committee number is: 2012/WN/Minel/3. We followed the European guidelines (European directives EU 86/609-STE123 and 2010/63/EU) for animal handling. To minimize the risk of infection of mosquito borne diseases, an internal net was used to protect the cattle against mosquito bites.

## Electronic supplementary material

Supplementary information

## Data Availability

All data generated or analysed during this study are included in this article.

## References

[1] Zug R, Hammerstein P (2012). Still a host of hosts for *Wolbachia*: analysis of recent data suggests that 40% of terrestrial arthropod species are infected. PLoS One.

[2] Klasson L (2008). Genome evolution of Wolbachia strain wPip from the Culex pipiens group. Mol Biol Evol.

[3] Laven H (1959). Speciation by cytoplasmic isolation in the Culex pipiens-complex. Cold Spring Harb Symp Quant Biol.

[4] Sinkins SP (2005). Wolbachia variability and host effects on crossing type in Culex mosquitoes. Nature.

[5] Dutton TJ, Sinkins SP (2004). Strain-specific quantification of Wolbachia density in Aedes albopictus and effects of larval rearing conditions. Insect Mol Biol.

[6] Sinkins SP, Braig HR, O’Neill SL (1995). Wolbachia pipientis: bacterial density and unidirectional cytoplasmic incompatibility between infected populations of Aedes albopictus. Exp Parasitol.

[7] Laven H (1967). Eradication of Culex pipiens fatigans through cytoplasmic incompatibility. Nature.

[8] O’Connor L (2012). Open release of male mosquitoes infected with a wolbachia biopesticide: field performance and infection containment. PLoS Negl Trop Dis.

[9] Brelsfoard CL, Dobson SL (2011). Wolbachia effects on host fitness and the influence of male aging on cytoplasmic incompatibility in Aedes polynesiensis (Diptera: Culicidae). J Med Entomol.

[10] Zhang D, Zheng X, Xi Z, Bourtzis K, Gilles JR (2015). Combining the sterile insect technique with the incompatible insect technique: I-impact of wolbachia infection on the fitness of triple- and double-infected strains of Aedes albopictus. PLoS One.

[11] Teixeira L, Ferreira A, Ashburner M (2008). The bacterial symbiont Wolbachia induces resistance to RNA viral infections in Drosophila melanogaster. PLoS Biol.

[12] Hedges LM, Brownlie JC, O’Neill SL, Johnson KN (2008). Wolbachia and virus protection in insects. Science (80-.)..

[13] Bian G, Xu Y, Lu P, Xie Y, Xi Z (2010). The endosymbiotic bacterium Wolbachia induces resistance to dengue virus in Aedes aegypti. PLoS Pathog.

[14] Moreira LA (2009). A Wolbachia symbiont in Aedes aegypti limits infection with dengue, Chikungunya, and Plasmodium. Cell.

[15] Walker T (2011). The wMel Wolbachia strain blocks dengue and invades caged Aedes aegypti populations. Nature.

[16] Joubert D. Albert, Walker Thomas, Carrington Lauren B., De Bruyne Jyotika Taneja, Kien Duong Hue T., Hoang Nhat Le Thanh, Chau Nguyen Van Vinh, Iturbe-Ormaetxe Iñaki, Simmons Cameron P., O’Neill Scott L. (2016). Establishment of a Wolbachia Superinfection in Aedes aegypti Mosquitoes as a Potential Approach for Future Resistance Management. PLOS Pathogens.

[17] Iturbe-Ormaetxe I, Walker T, O’ Neill SL (2011). Wolbachia and the biological control of mosquito-borne disease. EMBO Rep.

[18] Xi Z, Khoo CC, Dobson SL (2005). Wolbachia establishment and invasion in an Aedes aegypti laboratory population. Science (80-.)..

[19] McMeniman CJ (2009). Stable introduction of a life-shortening Wolbachia infection into the mosquito Aedes aegypti. Science (80-.)..

[20] Ant TH, Herd CS, Geoghegan V, Hoffmann AA, Sinkins SP (2018). The Wolbachia strain wAu provides highly efficient virus transmission blocking in Aedes aegypti. PLOS Pathog..

[21] Fraser Johanna E., De Bruyne Jyotika Taneja, Iturbe-Ormaetxe Iñaki, Stepnell Justin, Burns Rhiannon L., Flores Heather A., O’Neill Scott L. (2017). Novel Wolbachia-transinfected Aedes aegypti mosquitoes possess diverse fitness and vector competence phenotypes. PLOS Pathogens.

[22] Hoffmann AA (2011). Successful establishment of Wolbachia in Aedes populations to suppress dengue transmission. Nature.

[23] Frentiu Francesca D., Zakir Tasnim, Walker Thomas, Popovici Jean, Pyke Alyssa T., van den Hurk Andrew, McGraw Elizabeth A., O'Neill Scott L. (2014). Limited Dengue Virus Replication in Field-Collected Aedes aegypti Mosquitoes Infected with Wolbachia. PLoS Neglected Tropical Diseases.

[24] O'Neill Scott L., Ryan Peter A., Turley Andrew P., Wilson Geoff, Retzki Kate, Iturbe-Ormaetxe Inaki, Dong Yi, Kenny Nichola, Paton Christopher J., Ritchie Scott A., Brown-Kenyon Jack, Stanford Darren, Wittmeier Natalie, Anders Katherine L., Simmons Cameron P. (2018). Scaled deployment of Wolbachia to protect the community from Aedes transmitted arboviruses. Gates Open Research.

[25] Ferguson NM (2015). Modeling the impact on virus transmission of Wolbachia-mediated blocking of dengue virus infection of Aedes aegypti. Sci Transl Med.

[26] Aliota, M. T., Peinado, S. A., Velez, I. D. & Osorio, J. E. The wMel strain of Wolbachia Reduces Transmission of Zika virus by Aedes aegypti. *Sci*. *Rep*. **6** (2016). 10.1038/srep28792 PMC4929456 27364935

[27] van den Hurk AF (2012). Impact of Wolbachia on infection with chikungunya and yellow fever viruses in the mosquito vector Aedes aegypti. PLoS Negl Trop Dis.

[28] Dutra HLC (2016). Wolbachia Blocks Currently Circulating Zika Virus Isolates in Brazilian Aedes aegypti Mosquitoes. Cell Host and Microbe.

[29] Mousson L (2012). The native Wolbachia symbionts limit transmission of dengue virus in Aedes albopictus. PLoS Negl Trop Dis.

[30] Glaser RL, Meola MA (2010). The native Wolbachia endosymbionts of Drosophila melanogaster and Culex quinquefasciatus increase host resistance to West Nile virus infection. PLoS One.

[31] Joanne Sylvia, Vythilingam Indra, Teoh Boon-Teong, Leong Cherng-Shii, Tan Kim-Kee, Wong Meng-Li, Yugavathy Nava, AbuBakar Sazaly (2017). Vector competence of Malaysian Aedes albopictus with and without Wolbachia to four dengue virus serotypes. Tropical Medicine & International Health.

[32] Ahmad Noor Afizah, Vythilingam Indra, Lim Yvonne A. L., Zabari Nur Zatil Aqmar M., Lee Han Lim (2016). Detection of Wolbachia in Aedes albopictus and Their Effects on Chikungunya Virus. The American Journal of Tropical Medicine and Hygiene.

[33] Gloria-Soria A, Chiodo TG, Powell JR (2018). Lack of Evidence for Natural Wolbachia Infections in Aedes aegypti (Diptera: Culicidae). J. Med. Entomol..

[34] Coon KL, Brown MR, Strand MR (2016). Mosquitoes host communities of bacteria that are essential for development but vary greatly between local habitats. Mol. Ecol..

[35] Rossi P (2015). Mutual exclusion of Asaia and Wolbachia in the reproductive organs of mosquito vectors. Parasit Vectors.

[36] De Freece C (2014). Detection and isolation of the α-proteobacterium Asaia in Culex mosquitoes. Med. Vet. Entomol..

[37] Tantely ML, Le Goff G, Boyer S, Fontenille D (2016). An updated checklist of mosquito species (Diptera: Culicidae) from Madagascar. Parasite.

[38] Brunhes Jacques, Boussès Philippe, Tantely Michael Luciano, Kengne Pierre (2017). Un nouveau genre de Culicidae (Diptera), Paulianius n. gen., avec la description de trois nouvelles espèces malgaches. Annales de la Société entomologique de France (N.S.).

[39] Tantely LM, Boyer S, Fontenille D (2015). Review article: A review of mosquitoes associated with Rift Valley fever virus in Madagascar. American Journal of Tropical Medicine and Hygiene.

[40] Larrieu S (2013). Case report: A fatal neuroinvasive West Nile virus infection in a traveler returning from Madagascar: Clinical, epidemiological and veterinary investigations. Am. J. Trop. Med. Hyg..

[41] Kumar N. Pradeep, Rajavel A. R., Natarajan R., Jambulingam P. (2007). DNA Barcodes Can Distinguish Species of Indian Mosquitoes (Diptera: Culicidae). Journal of Medical Entomology.

[42] Atyame CM, Delsuc F, Pasteur N, Weill M, Duron O (2011). Diversification of Wolbachia endosymbiont in the Culex pipiens mosquito. Mol Biol Evol.

[43] Zittra, C. *et al*. Ecological characterization and molecular differentiation of Culex pipiens complex taxa and Culex torrentium in eastern Austria. *Parasites and Vectors* **9** (2016). 10.1186/s13071-016-1495-4 PMC4828795 27067139

[44] Smith JL, Fonseca DM (2004). Rapid assays for identification of members of the Culex (Culex) pipiens complex, their hybrids, and other sibling species (Diptera: culicidae). Am J Trop Med Hyg.

[45] Baldo L (2006). Multilocus sequence typing system for the endosymbiont Wolbachia pipientis. *Appl Env*. Microbiol.

[46] Zhou W, Rousset F, O’Neil S (1998). Phylogeny and PCR-based classification of Wolbachia strains using wsp gene sequences. Proc Biol Sci.

[47] Lo N, Casiraghi M, Salati E, Bazzocchi C, Bandi C (2002). How many wolbachia supergroups exist?. Mol Biol Evol.

[48] Stahlhut JK (2010). The mushroom habitat as an ecological arena for global exchange of Wolbachia. Mol Ecol.

[49] Gerth, M., Gansauge, M. T., Weigert, A. & Bleidorn, C. Phylogenomic analyses uncover origin and spread of the Wolbachia pandemic. *Nat*. *Commun*., 10.1038/ncomms6117 (2014). 10.1038/ncomms6117 25283608

[50] Fontenille D, Jupp PG (1989). The presence of the Culex (Culex) neavei mosquito in Madagascar, its relevance in the transmission of arboviruses. Arch Inst Pasteur Madagascar.

[51] Quan PL (2010). Moussa virus: a new member of the Rhabdoviridae family isolated from Culex decens mosquitoes in Cote d’Ivoire. Virus Res.

[52] Maquart M (2016). High Prevalence of West Nile Virus in Domestic Birds and Detection in 2 New Mosquito Species in Madagascar. PLoS One.

[53] Tantely Luciano Michaël, Cêtre-Sossah Catherine, Rakotondranaivo Tsiriniaina, Cardinale Eric, Boyer Sébastien (2017). Population dynamics of mosquito species in a West Nile virus endemic area in Madagascar. Parasite.

[54] Woolfit M, Iturbe-Ormaetxe I, McGraw EA, O’Neill SL (2009). An ancient horizontal gene transfer between mosquito and the endosymbiotic bacterium Wolbachia pipientis. Mol Biol Evol.

[55] Klasson L, Kambris Z, Cook PE, Walker T, Sinkins SP (2009). Horizontal gene transfer between Wolbachia and the mosquito Aedes aegypti. BMC Genomics.

[56] Hotopp J. C. D., Clark M. E., Oliveira D. C. S. G., Foster J. M., Fischer P., Torres M. C. M., Giebel J. D., Kumar N., Ishmael N., Wang S., Ingram J., Nene R. V., Shepard J., Tomkins J., Richards S., Spiro D. J., Ghedin E., Slatko B. E., Tettelin H., Werren J. H. (2007). Widespread Lateral Gene Transfer from Intracellular Bacteria to Multicellular Eukaryotes. Science.

[57] Fontenille D (1989). Transmission cycles of arboviruses in Madagascar. Arch Inst Pasteur Madagascar.

[58] Kittayapong P, Baisley KJ, Baimai V, O’Neill SL (2000). Distribution and diversity of Wolbachia infections in Southeast Asian mosquitoes (Diptera: Culicidae). J. Med. Entomol..

[59] Osei-Poku J, Han C, Mbogo CM, Jiggins FM (2012). Identification of Wolbachia strains in mosquito disease vectors. PLoS One.

[60] Nugapola, N. W. N. P., De Silva, W. A. P. P. & Karunaratne, S. H. P. P. Distribution and phylogeny of Wolbachia strains in wild mosquito populations in Sri Lanka. *Parasites and Vectors* **10** (2017). 10.1186/s13071-017-2174-9 PMC5424329 28490339

[61] Atyame CM (2015). Stable coexistence of incompatible Wolbachia along a narrow contact zone in mosquito field populations. Mol Ecol.

[62] Dumas E (2013). Population structure of Wolbachia and cytoplasmic introgression in a complex of mosquito species. BMC Evol Biol.

[63] Karami M (2016). Wolbachia endobacteria in natural populations of Culex pipiens of Iran and its phylogenetic congruence. J. Arthropod. Borne. Dis..

[64] LEGGEWIE M., KRUMKAMP R., BADUSCHE M., HEITMANN A., JANSEN S., SCHMIDT-CHANASIT J., TANNICH E., BECKER S. C. (2017). Culex torrentium mosquitoes from Germany are negative for Wolbachia. Medical and Veterinary Entomology.

[65] Raharimalala FN, Boukraa S, Bawin T, Boyer S, Francis F (2016). Molecular detection of six (endo-) symbiotic bacteria in Belgian mosquitoes: first step towards the selection of appropriate paratransgenesis candidates. Parasitol. Res..

[66] Atyame CM (2011). Cytoplasmic incompatibility as a means of controlling Culex pipiens quinquefasciatus mosquito in the islands of the south-western Indian Ocean. PLoS Negl Trop Dis.

[67] Rodhain F, Perez C, Ranaivosata J, Clerc Y, Coulanges P (1980). Rapport de mission entomologique sur les arbovirus en 1979. Arch. l’Institut Pasteur Madagascar.

[68] Andriamandimby SF (2010). Rift valley fever during rainy seasons, Madagascar, 2008 and 2009. Emerg. Infect. Dis..

[69] Jup PG (2002). The2000 epidemic of Rift Valley fever in Saudi Arabia: mosquito vector studies. Med Vet Entomol.

[70] Tantely ML (2013). Biology of mosquitoes that are potential vectors of Rift Valley Fever virus in different biotopes of the central highlands of Madagascar. J. Med. Entomol..

[71] Jeffries CL, Walker T (2016). Wolbachia Biocontrol Strategies for Arboviral Diseases and the Potential Influence of Resident Wolbachia Strains in Mosquitoes. Curr. Trop. Med. Reports.

[72] Silva Jéssica Barreto Lopes, Magalhães Alves Debora, Bottino-Rojas Vanessa, Pereira Thiago Nunes, Sorgine Marcos Henrique Ferreira, Caragata Eric Pearce, Moreira Luciano Andrade (2017). Wolbachia and dengue virus infection in the mosquito Aedes fluviatilis (Diptera: Culicidae). PLOS ONE.

[73] Favia G (2007). Bacteria of the genus Asaia stably associate with Anopheles stephensi, an Asian malarial mosquito vector. Proc Natl Acad Sci USA.

[74] Minard G (2013). Prevalence, genomic and metabolic profiles of Acinetobacter and Asaia associated with field-caught Aedes albopictus from Madagascar. FEMS Microbiol Ecol.

[75] Valiente Moro C, Tran FH, Raharimalala FN, Ravelonandro P, Mavingui P (2013). Diversity of culturable bacteria including Pantoea in wild mosquito Aedes albopictus. BMC Microbiol.

[76] Zouache K (2011). Bacterial diversity of field-caught mosquitoes, Aedes albopictus and Aedes aegypti, from different geographic regions of Madagascar. FEMS Microbiol Ecol.

[77] Jeffries CL (2018). Novel Wolbachia strains in Anopheles malaria vectors from Sub-Saharan Africa [version 1; referees: awaiting peer review]. Wellcome Open Res..

[78] Walker T, Song S, Sinkins SP (2009). Wolbachia in the Culex pipiens group mosquitoes: introgression and superinfection. J Hered.

[79] Werren JH, Windsor DM (2000). Wolbachia infection frequencies in insects: evidence of a global equilibrium?. Proc Biol Sci.

[80] de Oliveira C.D., Gonçalves D.S., Baton L.A., Shimabukuro P.H.F., Carvalho F.D., Moreira L.A. (2015). Broader prevalence of Wolbachia in insects including potential human disease vectors. Bulletin of Entomological Research.

[81] Yamada Y (2000). *Asaia bogorensis* gen. nov., sp. nov., an unusual acetic acid bacterium in the alpha-Proteobacteria. Int J Syst Evol Microbiol.

[82] Kumar S, Stecher G, Tamura K (2016). MEGA7: Molecular Evolutionary Genetics Analysis Version 7.0 for Bigger Datasets. Mol. Biol. Evol..

[83] Jolley, K. A., Chan, M. S. & Maiden, M. C. J. mlstdbNet - Distributed multi-locus sequence typing (MLST) databases. *BMC Bioinformatics* **5** (2004). 10.1186/1471-2105-5-86 PMC459212 15230973

[84] Tamura K, Nei M (1993). Estimation of the number of nucleotide substitutions in the control region of mitochondrial DNA in humans and chimpanzees. Mol. Biol. Evol..

