# Supplementary material for: Diverse novel resident Wolbachia strains in Culicine mosquitoes from Madagascar
Source: Sci Rep. 2018 Nov 29;8:17456. doi: 10.1038/s41598-018-35658-z (PMC6265278; doi:10.1038/s41598-018-35658-z)
Supplement: Supplementary file 1 — Supplementary information [file 41598_2018_35658_MOESM1_ESM.pdf]

Supplementary information

Diverse novel resident *Wolbachia* strains in Culicine mosquitoes from Madagascar

Claire L Jeffries, Luciano M Tantely, Fara N Raharimalala, Eliot Hurn, Sébastien Boyer & Thomas Walker

Supplementary table S1. Additional sample details and CO1 GenBank accession numbers

Supplementary table S2. Additional sample details and *Wolbachia* 16S, wsp and MLST gene GenBank accession numbers.

Supplementary table S3. Summary of qualitative characteristics, median humidity, median temperature and altitude of collection sites.

Supplementary table S4. Arbovirus screening assays including PCR primer/probes sequences and cycling conditions used to screen mosquitoes.

Supplementary table S1. Additional sample details and CO1 GenBank accession numbers. The sample code, location and species are shown, in addition to the *Wolbachia* / RVFV status and GenBank CO1 accession number.

**Sample ID**	**Alternative ID**	**Location**	**Species**	***Wolbachia* / RVFV status**	**CO1 accession number**
TSA-AMAD-1	Aedo. madagascarica (W+)	Tsaramandroso	*Aedeomyia madagascarica*	W+	MK033247
TSA-CANT-1	Cx. antennatus (W+) (RVFV+)	Tsaramandroso	*Culex antennatus*	W+ / RVFV+	MK033248
TSA-CDEC-1	Cx. decens (W+) 1	Tsaramandroso	*Culex decens*	W+	MK033249
TSA-CDEC-2	Cx. decens (W+) 2	Tsaramandroso	*Culex decens*	W+	MK033250
TSA-CDEC-3	Cx. decens (W+) 3	Tsaramandroso	*Culex decens*	W+	MK033251
TSA-CDUT-1	Cx. duttoni (W+)	Tsaramandroso	*Culex duttoni*	W+	MK033252
TSA-CTRI-1	Cx. tritaeniorhynchus (RVFV+)	Tsaramandroso	*Culex tritaeniorhynchus*	W- / RVFV+	MK033253
TSA-MUNI-1	Man. uniformis (W+)	Tsaramandroso	*Mansonia uniformis*	W+	MK033254
TSA-USP1-1	Ura. sp. 1 (W+)	Tsaramandroso	*Uranotaenia* sp. 1	W+	MK033255
TSA-USP2-1	Ura. sp. 2 (W+) 1	Tsaramandroso	*Uranotaenia* sp. 2	W+	MK033256
TSA-USP2-2	Ura. sp. 2 (W+) 2	Tsaramandroso	*Uranotaenia* sp. 2	W+	MK033257
CU1-ANK-CSP1-1	Culex sp. 1 (W-) 1	Ankazobe	*Culex* sp. 1	W-	MK033258
CU2-ANK-CUNI-1	Cx. univittatus (W-) 1	Ankazobe	*Culex univittatus*	W-	MK033259
CU3-ANK-CPIP-1	Cx. pipiens (W-) 1	Ankazobe	*Culex pipiens*	W-	MK033260
CU4-ANK-CUNI-2	Cx. univittatus (W-) 2	Ankazobe	*Culex univittatus*	W-	MK033261
CU5-ANK-CUNI-3	Cx. univittatus (W-) 3	Ankazobe	*Culex univittatus*	W-	MK033262
CU6-ANK-CSP1-2	Culex sp. 1 (W-) 2	Ankazobe	*Culex* sp. 1	W-	MK033263
CU7-TSA-CSP1-1	Culex sp. 1 (W-) 3	Tsaramandroso	*Culex* sp. 1	W-	MK033264
CU8-TSA-CPIP-1	Cx. pipiens (W-) 2	Tsaramandroso	*Culex pipiens*	W-	MK033265
CU9-ANK-CPIP-2	Cx. pipiens (W-) 3	Ankazobe	*Culex pipiens*	W-	MK033266
CU10-ANK-CPIP-3	Cx. pipiens (W-) 4	Ankazobe	*Culex pipiens*	W-	MK033267
CU11-TSA-CPIP-2	Cx. pipiens (W-) 5	Tsaramandroso	*Culex pipiens*	W-	MK033268
CU12-IVA-CPIP-1	Cx. pipiens (W-) 6	Ivato Aeroport	*Culex pipiens*	W-	MK033269

Supplementary table S2. Additional sample details and *Wolbachia* 16S, wsp and MLST gene GenBank accession numbers. Sample codes, *Wolbachia* strain names and *Wolbachia* MLST gene sequence GenBank accession numbers.

**Sample ID**	**Location**	**Host species**	**Strain**	**16S**	**wsp**	**gatB**	**coxA**	**hcpA**	**ftsZ**	**fbpA**
TSA-AMAD-1	Tsaramandroso	*Aedeomyia madagascarica*	wMad	MK026554	MK033270	MK033279	MK033288	MK033297	MK033305	MK033312
TSA-AMAD-2	Tsaramandroso	*Aedeomyia madagascarica*	wMad	-	MK033271	-	-	-	-	-
TSA-AMAD-3	Tsaramandroso	*Aedeomyia madagascarica*	wMad	-	MK033272	-	-	-	-	-
TSA-CANT-1	Tsaramandroso	*Culex antennatus*	wAnt	MK026555	-	-	-	-	-	-
TSA-CDEC-1	Tsaramandroso	*Culex decens*	wDec	MK026556	MK033273	MK033280	MK033289	MK033298	-	MK033313
TSA-CDEC-2	Tsaramandroso	*Culex decens*	wDec	MK026557	MK033274	MK033281	MK033290	MK033299	MK033306	MK033314
TSA-CDUT-1	Tsaramandroso	*Culex duttoni*	wDutt	MK026558	-	-	-	-	-	-
TSA-FCIR-1	Tsaramandroso	*Ficalbia circumtestacea*	wCir	-	-	MK033282	MK033291	MK033300	MK033307	MK033315
TSA-MUNI-1	Tsaramandroso	*Mansonia uniformis*	wUnif-Mad	MK026559	-	MK033283	MK033292	-	MK033308	MK033316
ANI-USP1-1	Anivorano Nord	*Uranotaenia* sp. 1	wUra1	MK026560	MK033275	MK033284	MK033293	MK033301	MK033309	MK033317
TSA-USP1-1	Tsaramandroso	*Uranotaenia* sp. 1	wUra1	MK026561	MK033276	MK033285	MK033294	MK033302	MK033310	MK033318
TSA-USP2-1	Tsaramandroso	*Uranotaenia* sp. 2	wUra2	MK026562	MK033277	MK033286	MK033295	MK033303	MK033311	MK033319
TSA-USP2-2	Tsaramandroso	*Uranotaenia* sp. 2	wUra2	MK026563	MK033278	MK033287	MK033296	MK033304	MK033307	MK033320

Supplementary table S3. Summary of qualitative characteristics, median humidity, median temperature and altitude of collection sites.

Commune	Village	Nearest water bodies	Natural flora	Natural fauna	Agriculture	Animal husbandry	Construction / housing	Human population density	Median humidity (25th percentile, 75th percentile)	Median temperature (25th percentile, 75th percentile)	Altitude (meters above sea level)
Anivorano Nord	Anivorano III	river, swamp	forested areas	cats, dogs	n/a	zebu, poultry	thatched roofs, sheet metal walls	sparsely populated	74 (65, 83)	20.5 (19.5, 22.5)	357
Tsaramandroso	Ambomiharina	lake	scattered trees, bushes	dogs	Rice paddies, swede, cane	zebu, goats	thatched roofs, wood walls	populated	57 (53,70)	24.0 (21.5, 28.0)	84
Bemokotra	Antafia	lake	trees, scattered shrubbery	bats, cats	cane	zebu, poultry, swine	concrete houses/wood walls thatched roofs	populated	50 (40, 60)	26.0 (23.5, 29.5)	64
Ankazobe	Ambohimarina	n/a (large city)	patches of dense greenery	cats	n/a	zebus, poultry, swine	clay walls/thatched roofs, brick walls/sheet metal roofs	population	79 (63, 90)	14.5 (13.0, 18.5)	1212
Ivato Aeroport	Ivato Imerimandroso	canals, rice paddies	grassland	birds	Rice paddies, banana plantation	zebu, poultry	concrete houses	sparsely populated	88 (61, 100)	11.0 (9.0, 15.5)	1261

Supplementary table S4. Arbovirus screening assays including PCR primer/probes sequences and cycling conditions used to screen mosquitoes.

Arbovirus	Primer and probe sequences	Cycling conditions	Reference
DENV	5′-TTGAGTAAACYRTGCTGCCTGTAGCTC-3<br>5′-GAGACAGCAGGATCTCTGGTCTYTC-3′	95°C for 10 min<br>50 cycles: 95°C for 10 sec, 60°C for 10 sec, 72°C for 10 sec	[1]
ZIKV	5'- CCGCTGCCCAACACAAG -3'<br>5'- CCACTAACGTTCTTTTGCAGACAT -3'<br>5'-FAM-AGCCTACCTTGACAAGCAGTCAGACACTCAA-TAMRA3'	95°C for 10 min<br>45 cycles: 95°C for 10 sec, 52°C for 30 sec	[2]
CHIKV	5'- CTCATA CCGCATCCGCATCAG-3'<br>5'- ACATTGGCCCCACAAT GAATTTG-3'	95°C for 10 min<br>40 cycles: 95°C for 10 sec, 56°C for 10 sec, 72°C for 15 sec	[3]
WNV	5'-CCTGTGTGAGCTGACAAACTTAGT-3'<br>5'-GCGTTTTAGCATATTGACAGCC-3'	95°C for 10 min<br>45 cycles: 95°C for 10 sec, 60°C for 10 sec, 72°C for 20 sec	[4]
YFV	5'- AATCGAGTTGCTAGGCAATAAACAC-3'<br>5'- TCCCTGAGCTTTACGACCAGA-3'	95°C for 10 min<br>40 cycles: 95°C for 10 sec, 58°C for 10 sec, 72°C for 10 sec	[5]
RVFV	5'-CTAGCCGTTTCACAAACTGGG-3'<br>5'-GACTGARGAYTCTGAATTGCACC-3'	95°C for 10 min<br>45 cycles: 95°C for 10 sec, 60°C for 10 sec, 72°C for 20 sec	[6]

1. Lai YL, Chung YK, Tan HC, Yap HF, Yap G, Ooi EE, et al. Cost-effective real-time reverse transcriptase PCR (RT-PCR) to screen for Dengue virus followed by rapid single-tube multiplex RT-PCR for serotyping of the virus. J Clin Microbiol. 2007/01/12. 2007;45: 935–941. doi:10.1128/JCM.01258-06
2. Lanciotti RS, Kosoy OL, Laven JJ, Velez JO, Lambert AJ, Johnson AJ, et al. Genetic and serologic properties of Zika virus associated with an epidemic, Yap State, Micronesia, 2007. Emerg Infect Dis. 2008;14: 1232–1239. doi:10.3201/eid1408.080287
3. Ali UH, Vasan SS, Thayan R, Angamuthu C, Lim LH, Sekaran SD. Development and evaluation of a one-step SYBR-Green I-based real-time RT-PCR assay for the detection and quantification of Chikungunya virus in human, monkey and mosquito samples. Trop Biomed. 2010;27: 611–623.
4. Linke S, Ellerbrok H, Niedrig M, Nitsche A, Pauli G. Detection of West Nile virus lineages 1 and 2 by real-time PCR. J Virol Methods. 2007;146: 355–358. doi:10.1016/j.jviromet.2007.05.021
5. Dash PK, Boutonnier A, Prina E, Sharma S, Reiter P. Development of a SYBR green I based RT-PCR assay for yellow fever virus: application in assessment of YFV infection in Aedes aegypti. Virol J. 2012/01/24. 2012;9: 27. doi:10.1186/1743-422X-9-27
6. Maquart M, Temmam S, Héraud J-M, Leparc-Goffart I, Cêtre-Sossah C, Dellagi K, et al. Development of real-time RT-PCR for the detection of low concentrations of Rift Valley fever virus. J Virol Methods. 2014;195: 92–99. doi:10.1016/j.jviromet.2013.10.001.
